# Biotechnology in Environmental Monitoring and Pollution Abatement

**DOI:** 10.1155/2014/235472

**Published:** 2014-04-23

**Authors:** Kannan Pakshirajan, Eldon R. Rene, Aiyagari Ramesh

**Affiliations:** ^1^Department of Biotechnology, Indian Institute of Technology Guwahati, Guwahati 781039, Assam, India; ^2^Pollution Prevention and Resource Recovery Chair Group, Department of Environmental Engineering and Water Technology, UNESCO-IHE Institute for Water Education, Westvest 7, 2611 AX Delft, The Netherlands

In recent years, the demand for the use of sustainable and eco-friendly environmental processes is rapidly growing subjected to economic, public, and legislation pressure. Biotechnology provides a plethora of opportunities for effectively addressing issues pertaining to the monitoring, assessment, modeling, and treatment of contaminated water, air, and solid waste streams. In this context, source tracking of environmental pollutants and process modeling using biological based methods are becoming increasingly important, mainly owing to the accuracy and robustness of such techniques. The different biotechniques available nowadays, thus, represent both well-established and novel (bio)technologies, although several aspects of their performance are still to be tested. For instance, the use of novel biocatalysts and reactor designs, the understanding of microbial community dynamics and mechanisms occurring within a (bio)reactor, and the assessment of the performance of (bio)reactors during long-term operation and its modeling. If these mechanisms are understood and the barriers are overcome, novel biotechniques will potentially change the way users rebuild technologies for the sustainable use of different biological processes for wastewater, air, and solid waste treatment.

This special issue received 34 research/review articles over a period of 6 months, of which 21 high-quality papers (62%) were accepted for publication following a double blind peer-review process. These accepted papers focus on the various fundamental and applied engineering aspects of different techniques and processes that have potential practical implications in emerging fields of environmental biotechnology. This special issue highlights certain challenging issues pertaining to environmental monitoring and pollution abatement that can be categorized into five thematic research areas.

*Environmental Monitoring and Modeling*. In developing countries, water, air, and soil pollution has become a persisting environmental problem due to rapid industrialization and urbanization. Using environmental Kuznets curve (EKC) it was observed that, during early stages of economic development in a particular region, the environment paid a high price for economic growth as the human race used technology to exploit all possible valuable resources. Nevertheless, in agricultural areas, N, P, and K compounds are easily transported by farmland drainage and surface water to valuable water resources resulting in the deterioration of water quality that warrants the use of novel biosensors to monitor water quality. Recently, it has been proposed that cellular-based biosensor technologies, that is, the bioelectric recognition assay (BERA), utilize live, functional cells in a gel matrix coupled with a sensor system that is able to measure changes in the cellular electric properties. Cells that are able to specifically interact with a target analyte produce a unique pattern of electrical potential as a result of their interaction with this analyte.

Concerning modeling, traditionally, the performance of many bioprocesses [1] has been modeled/predicted using process-based models that are based on mass balance principles, simple reaction kinetics, and a plug flow of water/air stream. An alternate modeling procedure consists of a data driven approach wherein the principles of artificial intelligence (AI) are applied with the help of neural networks [2, 3]. The concept of neural network modeling has widespread applications in the fields of applied biosciences and bioengineering. The following research papers (1–3) were accepted under this category.

1. * “Environmental Kuznets curve analysis of the economic development and nonpoint source pollution in the Ningxia Yellow River irrigation districts in China”* by C. Mao et al.
2. * “Back propagation neural network model for predicting the performance of immobilized cell biofilters handling gas-phase hydrogen sulphide and ammonia”* by E. R. Rene et al.
3. * “Pesticide residue screening using a novel artificial neural network combined with a bioelectric cellular biosensor”* by K. P. Ferentinos et al.

*Pollutant Removal and Toxicity*. Environmental pollutants such as heavy metals and pesticides are commonly present in water emanating from acid mine drainage or other industries and from agricultural runoffs. These toxic pollutants can accumulate in living organisms and produce adverse effect such as carcinogenicity and acute toxicity. Complete mineralization and/or removal of these pollutants and their toxic byproducts can be achieved using biological process that uses active bacterial/fungal/mixed microbial cultures [4]. Microbial consortia have been shown to be more suitable for bioremediation of recalcitrant compounds such as pesticide residues, as their biodiversity supports environmental survival and increases the number of catabolic pathways available for contaminant biodegradation. In the case of heavy metal contaminated wastewaters, biosorption has emerged as a promising low-cost methodology wherein biological catalysts are employed to remove and recover heavy metals from aqueous solutions [5]. The metal removal mechanism is a complex process that depends on the chemistry of metal ions, cell wall compositions of microorganisms, physiology of the organism, and physicochemical factors like pH, temperature, time, ionic strength, and metal concentration. The following papers (4–8) were selected for publication under this section of the special issue.

(4) * “Dissolution of arsenic minerals mediated by dissimilatory arsenate reducing bacteria: estimation of the physiological potential for arsenic mobilization”* by D. Lukasz et al.
(5) * “Kinetics of molybdenum reduction to molybdenum blue by Bacillus sp. strain A. rzi” *by A. R. Othman et al.
(6) * “The uptake mechanism of Cd(II), Cr(VI), Cu(II), Pb(II), and Zn(II) by mycelia and fruiting bodies of Galerina vittiformis” *by D. Damodaran et al.
(7) * “Toxicity of superparamagnetic iron oxide nanoparticles on green alga Chlorella vulgaris”* by L. Barhoumi and D. Dewez.
(8) * “Enhanced removal of a pesticides mixture by single cultures and consortia of free and immobilized Streptomyces strains”* by M. S. Fuentes et al.

*Biofuels Production*. Biohydrogen production through anaerobic fermentation is a sustainable alternative for managing the recent (dogging) energy crisis and creating a sustainable green environment. Fermentative hydrogen production processes are technically feasible and economically cost-competitive and have large-scale commercialization implications [6, 7]. Besides some of the pure microbial species, that can be used to produce biofuels, as of late, it was shown that microbes present in the sediments of mangroves have the capability to yield biohydrogen. Mangrove sediments are inherently rich in organic content and offer the following advantages: flexible substrate utilization and the simplicity of handling, no major storage problems, minimal preculturing requirements, and sediments being available at low cost. Alternatively, the development of (bio)energy using marine and freshwater microalgae as a 3rd generation biomass feedstock has also been explored recently because microalgae can grow fast with high specific growth rates and have excellent CO_2_ absorption capacity and better regulation of lipid and sugar content under various culture conditions. Microalgae exhibit a high photosynthetic efficiency and a strong capacity to adapt to the environment (e.g., high salinity, heavy metal ion content, presence of toxicants, and high CO_2_ concentration). The following papers (9–11) describe the production of biohydrogen and biodiesel.

(9) * “Biohydrogen production and kinetic modeling using sediment microorganisms of Pichavaram mangroves, India”* by P. Mullai et al.
(10) * “Production of biodiesel from Chlorella sp. enriched with oyster shell extracts” *by C. S. Choi et al.
(11) * “Enhancement of biodiesel production from marine alga, Scenedesmus sp. through in situ transesterification process associated with acidic catalyst”* by G. V. Kim et al.

*Microbial Products for the Environment*. With increasing concern for the natural environment, biosynthetic and biodegradable biopolymers such as poly-*β*-hydroxybutyrate (PHB) have attracted great interest because of their excellent biodegradability and being environmentally benign and sustainable. The high production cost of PHB can be curtailed by strain development, improving fermentation and separation processes, and using inexpensive carbon source. Due to recent advancements in fermentation technology and allied sciences, alternative purification solutions are under investigation, among which microbiological ways of utilization of byproducts are very interesting and promising. Such a solution could result in better overall process productivity and facilitate the downstream processing. Concerning the use of enzymes, owing to its lignolytic enzyme system, the white-rot fungus* Phanerochaete chrysosporium* has been applied in many bioremediation studies [8]. Its ability to degrade a variety of pollutants is thus related to the production of lignin peroxidase and manganese peroxidase, two lignin-modifying enzymes generally expressed under nitrogen-limited culture conditions, as well as to the intracellular cytochrome P450 system. Another practical aspect worth highlighting in this section is the use of an enhanced biological phosphorus removal (EBPR) for phosphorus removal from wastewaters. In EBPR, alternative anaerobic and aerobic phases are adopted and polyphosphate accumulating organisms (PAOs) with excess phosphorus accumulation ability can be enriched. During the anaerobic phase, PAOs take up organic carbons such as acetate and propionate and store them as intracellular polymers such as PHBs, with polyphosphate as the energy source and glycogen as the reducing power source. The metabolism of PAOs and dynamics of polymers under different organic carbon concentrations deserves in-depth examination in order to elucidate the function of polymers in EBPR. Investigating the dynamics of polymers under endogenous respiration conditions will also provide solutions for controlling and adjusting the EBPR performance under low organic carbon induced shock conditions. The following papers (12–17) were accepted for publication under the theme of “microbial production for the environment.”

(12) * “Degradation of diuron by Phanerochaete chrysosporium: role of ligninolytic enzymes and cytochrome P450” * by J. da Silva Coelho-Moreira et al.
(13) * “Dynamics of intracellular polymers in enhanced biological phosphorus removal processes under different organic carbon concentrations”* by L. Xing et al.
(14) * “Microbial purification of postfermentation medium after 1,3-PD production from raw glycerol”* by D. Szymanowska-Powałowska et al.
(15) * “Rhizobium pongamiae sp. nov. from root nodules of Pongamia pinnata”* by V. Kesari et al.
(16) * “Persistent organic pollutants induced protein expression and immunocrossreactivity by Stenotrophomonas maltophilia PM102: a prospective bioremediating candidate” * by P. Mukherjee and P. Roy.
(17) * “Poly *β*-hydroxybutyrate production by Bacillus subtilis NG220 using sugar industry wastewater”* by G. Singh et al.

*Eco-Efficient Bioprocesses for the Environment*. Nutrient-rich wastewater streams when discharged into receiving water bodies often lead to undesirable problems such as algal blooms, eutrophication, and oxygen deficit. For such commonly reported situations in many developing countries, advanced treatment technologies cannot be applied to treat wastewater due to the requirement of high energy and skilled labor force, high operating and maintenance costs. Under such conditions, low-cost natural treatment systems can be effectively used not only for waste treatment, but also for conserving biological communities in poor nations of the world [9]. For the (bio)treatment of slaughterhouse wastewater, sequencing batch reactors (SBRs) were recommended as one of the best options because they are capable of removing organic carbon, nutrients, and suspended solids from wastewater in a single tank and also have low capital and operational costs. In order to maintain the long-term performance of bioreactors (e.g., stirred tank bioreactor), various strategies to improve the oxygen transfer in bioreactors have been proposed, for instance, dispersing a nonaqueous, organic, second liquid-phase that is immiscible to the system. The presence of this organic phase modifies the medium in such a way that it could carry more oxygen and this approach was found successful in the past. The organic phase has strong affinity for oxygen so that it can increase the apparent solubility of oxygen in water, which in turn increases the specific activity of microorganisms, yielding high removal of target pollutant in bioreactors. In this special issue, four papers (18–21) deal with the operational characteristics of natural and conventional bioprocesses and their advantages to treat specific industrial wastewaters.

(18) * “Enhancement of oxygen mass transfer and gas holdup using palm oil in stirred tank bioreactors with xanthan solutions as simulated viscous fermentation broths”* by S. M. Sauid et al.
(19) * “Treatment of slaughter house wastewater in a sequencing batch reactor: performance evaluation and biodegradation kinetics” * by P. Kundu et al.
(20) * “Performance study of chromium (VI) removal in presence of phenol in a continuous packed bed reactor by Escherichia coli isolated from East Calcutta wetlands” * by B. Chakraborty et al.
(21) * “Natural treatment systems as sustainable ecotechnologies for the developing countries”* by Q. Mahmood et al.

It is quite apparent from the discussions and conclusions made by the authors from the papers published in this special issue, as well as other recently published scientific literature related to environmental research, that there is an urgent need to translate most of the lab-based research into field-based research in order to witness sustainable solutions to persisting environmental problems. Future research should focus/address crucial issues pertaining to (i) biomarkers for environmental pollutants, (ii) development of new biosensors for environmental monitoring, (iii) development of new biocatalysts (bacteria, fungi, yeast, and algae) for environmental applications, (iv) development of innovative bioreactors for wastewater and air pollution control, and (v) studies on the socioeconomic implications and technological evaluation of new bioprocesses.

We firmly believe that the collection of papers presented in this special issue will stimulate interest within the global research community and would help peers in their research pursuits.
